# Setting up a new MRgFUS service for fibroids - the challenges and solutions

**DOI:** 10.1186/2050-5736-2-S1-A22

**Published:** 2014-12-10

**Authors:** Paul M Crowe, Shirin Irani

**Affiliations:** 1 Birmingham Fibroid Clinic, Spire Parkway Hospital, UK

## Purpose of the study

To discuss the practicalities of setting up MRgFUS service for uterine fibroids and in particular the challenges of formulating an effective business plan in a time of financial austerity and curtailment of health service budgets.

## Background

The authors, an interventional radiologist and a gynaecologist, have worked together closely for 10 years delivering a comprehensive service for the treatment of uterine fibroids in both the state and private health sectors. Despite recognising the clinical potential of MRgFUS and its undoubted appeal to patients we encountered difficulty in engaging any hospital partner to make the necessary capital investment.

## Materials and methods

We describe the business planning process and arguments put forward to support our strategy. Close interdisciplinary working between specialities is fundamental to success as is the nurturing of links in both healthcare management and industry. Challenges include not only the capital costs of equipment purchase but also the opportunity cost of lost scanning time on MRI scanners also used for diagnostic imaging. The requirement for a particular make of MRI scanner can also hinder business plan progress due to manufacturer representation within a particular national market. Reluctance of both government health services and private health insurers to reimburse innovative treatments is a further hindrance to overcome.

## Results

We share our experience of several unsuccessful and one ultimately successful bid to set up a service with a hospital partner and to provide MRgFUS as an addition to our repertoire of minimally invasive fibroid treatment options. We outline the ground work and business planning processes and arguments in terms of clinical quality, patient choice and health economics used to support our service development strategy.

## Conclusion

There are undoubtedly logistical and economic obstacles to overcome in setting up a new MRgFUS service but these are not insurmountable. With clarity of purpose, perseverance, an understanding of the health economic arguments and a level of persuasive skills these obstacles can be overcome.

